# Reliable measurement methods for the isovolumic relaxation time: comparisons of dual gate Doppler and seven other methods

**DOI:** 10.1007/s12574-023-00624-w

**Published:** 2023-09-25

**Authors:** Noriko Kimura, Hiroyuki Toide, Sayuki Kobayashi, Emi Tsujimoto, Asuka Honda, Kenta Sawada, Ayako Higashi, Yuri Koshikawa, Shinsei Hana, Yuji Itabashi

**Affiliations:** 1 Center of Medical Ultrasonics, Saitama Medical Center, Dokkyo Medical University, 2-1-50 Minamikoshigaya, Koshigaya City, Saitama 343-8555 Japan; 2 Department of Cardiology, Saitama Medical Center, Dokkyo Medical University, 2-1-50 Minamikoshigaya, Koshigaya City, Saitama 343-8555 Japan

**Keywords:** Isovolumic relaxation time, Diastolic function, Dual Gate Doppler, Continuous wave Doppler, Pulsed Doppler

## Abstract

**Purpose:**

Isovolumic relaxation time (IVRT) is a useful indicator of diastolic dysfunction. However, a measurement method for IVRT has not been established. The Dual Gate Doppler method, which can record two separate pulse-wave Doppler signals simultaneously using two sample gates, may be ideal for measuring IVRT. This study aimed to evaluate the accuracy of IVRT measured using conventional methods versus that measured using the Dual Gate Doppler method.

**Methods:**

A total of 104 patients (mean age 58 ± 21 years, 48 women) were examined using ultrasound equipment with Dual Gate Doppler at our hospital. In addition to Dual Gate Doppler method, IVRTs were measured using seven different methods: pulsed Doppler (PW method), continuous wave Doppler (CW method), and other methods. The IVRT values obtained using the Dual Gate Doppler method were compared with those measured using other methods.

**Results:**

All IVRTs measured using conventional methods showed a strong correlation with the that measured using the Dual Gate Doppler method. However, there were slight deifferences among the IVRTs depending on the method. The PW method and the PW time difference method using only the PW showed small statistical bias and were not complicated. The IVRT measured using the CW method was significantly longer than that measured using the Dual Gate Doppler method.

**Conclusions:**

Among the conventional methods, the PW method was the simplest and most practical method for measuring the IVRT in any conditions as arrhythmias. It is important to recognize the characteristics of IVRTs based on the measurement method.

## Introduction

With the aging of populations in developed countries, the importance of accurately estimating diastolic function in patients with heart failure has been increasing. Cardiac diastolic function is generally assessed based on the guidelines of the American Society of Echocardiography (ASE) and European Association of Cardiovascular Imaging (EACVI) [1]. In these guidelines, the ratio of early diastolic filling velocity of mitral flow to e’ of tissue velocity, e’, tricuspid regurgitation velocity, left atrial volume index, and ratio of early diastolic filling velocity to atrial filling velocity of mitral flow are considered important parameters for estimating diastolic function. However, patients with diastolic dysfunction often experience tachycardia or atrial fibrillation. Because some of these parameters cannot be measured in these patients, it is difficult to estimate diastolic dysfunction according to the algorithms in the guidelines. Based on this background, the isovolumic relaxation time (IVRT) is used as a reproducible and clinically valuable parameter to evaluate diastolic function, even in patients with arrhythmias, such as atrial fibrillation. These guidelines recommend calculating the IVRT by continuous-wave Doppler (CW) as the standard method, except in patients with restrictive cardiomyopathy [2–4]. In the field of restrictive cardiomyopathy, there are different studies on IVRTs measured using the pulsed Doppler (PW) method [5] or using both the second sound in the heart sound (IIs) and PW measurement [6]. The guidelines state that the IVRT measurements vary by measurement method; however, the details of their differences have not been clarified. The normal value of the IVRT is defined as < 70 ms using the CW method [1], which is prolonged or shortened depending on the presence or absence of elevated left ventricular filling pressure. In contrast, Dual Gate Doppler can display two separate sample gates that allow the recording of two PW measurements from different locations in the same cardiac cycle [7], and it can measure a more precise value directly without using technical complications. Some investigators also were reported about the usefulness and preciseness of Dual Gate Doppler for evaluating cardiac diastolic function [8, 9]. Therefore, an accurate IVRT can be measured using Dual Gate Doppler, and it can be used as a reliable reference for the validation of IVRTs measured using various conventional methods. We aimed to identify the most reliable IVRT measurement method by comparing the differences in IVRTs measured by each conventional method from that calculated by the Dual Gate Doppler method.

## Materials and methods

### Study design and population

A total of 104 participants (mean age, 58 ± 21 years; 48 women) were referred to undergo IVRT measurements at our institution from January 2020 to November 2020. The participants were as follows: 42 healthy volunteers, 15 with abnormal electrocardiography (ECG), 14 individuals with hypertension, 9 with diabetes mellitus, 4 with dyslipidemia, 15 with ischemic heart disease, 3 with cardiomyopathy, 5 with valvular diseases, and 2 with hemodialysis. This study had been approved by clinical research ethics review committee in our hospital and a prospective study. Only participants could be obtained the consent for this study were included.

Transthoracic echocardiography (TTE) was performed using an ultrasound machine (LISENDO 880; FUJIFILM Healthcare Corporation, Tokyo, Japan). We used a cardiac microphone (MA-300; Fukuda Denshi, Tokyo, Japan). In all cases, the cardiac microphone was placed at the site of maximum IIs. All participants were in sinus rhythm and were hemodynamically stable during the examination.

### Echocardiography

We measured IVRT using the Dual Gate Doppler method and seven other methods using the same machine at the same heart rate for all patients. These seven methods were described in the evaluation of left ventricular diastolic function of the ASE and EACVI guidelines [1] and in the reference literature of these guidelines [2–6]. The CW and PW methods were published in the ASE and EACVI guidelines for the IVRT measurement [1]. We combined phonocardiography (PCG), ECG, M-mode echocardiography, and Doppler echocardiography in the seven measurement methods of the IVRT as follows.

**Fig. 1:**
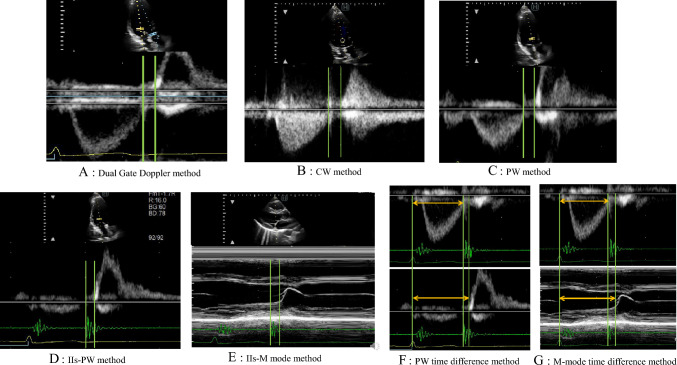
**A** Dual-gate Doppler method. **B** Continuous wave Doppler (CW) method. **C** Pulsed Doppler (PW) method. **D** Second sound-pulsed Doppler (IIs-PW) method. **E** Second sound-M mode (IIs-M mode) method. **F** PW time difference method. **G** M-mode time difference method. The isovolumic relaxation time (IVRT) is obtained by measuring the time between the green lines in Fig. 1A, B, C, D, and E. The IVRT is calculated by measuring from the top of the R-wave on electrocardiography to the end of left ventricular outflow (orange arrow) and the beginning of left ventricular inflow (orange arrow) in Fig. 1F and G

- (1) Dual Gate Doppler method (Fig. 1-A): Each sample volume was placed at the left ventricular outflow tract and the tip of the mitral valve during mitral valve opening. The time interval from the end of left ventricular outflow to the beginning of mitral inflow was measured at the apical three-chamber view of the left ventricle (LV) by Dual Gate Doppler.
- (2) CW method (Fig. 1-B): The cursor was placed on the line passing through the aortic mitral curtain in LV outflow tract to simultaneously displayed end of aortic ejection and onset of mitral inflow at the apical three chamber view of the LV. The time interval from the end of the left ventricular outflow to the beginning of the mitral inflow was measured using the CW method.
- (3) PW method (Fig. 1-C): The sample volume was placed at the tip of the anterior mitral leaflet on the line passing through the aortic mitral curtain at the apical three-chamber view of the LV. The time interval from the end of the left ventricular outflow to the beginning of the mitral inflow was measured using the PW method.
- (4) IIs-PW method (Fig. 1-D): IIs was recorded using PCG. The sample volume was placed between the tips of the anterior and posterior mitral leaflets. The time interval from the beginning of IIs to the beginning of mitral inflow was measured using the PW method.
- (5) IIs-M-mode method (Fig. 1-E): IIs was recorded by PCG. M-mode echocardiography was performed in the parasternal long-axis view. The time interval from the beginning of IIs to the beginning of the mitral opening phase, determined by point D in the M-mode record, was measured in the same heartbeat.
- (6) PW time difference method (Fig. 1-F): The first interval was between the time from the top of the R-wave on ECG to the end of left ventricular outflow by PW in the apical three-chamber view of the LV. The second interval was between the time from the top of the R-wave on ECG to the beginning of mitral valve inflow by PW. The time difference was calculated by subtracting the former time from the latter time.
- (7) M-mode time difference method (Fig. 1-G): The first interval was between the time from the top of the R-wave on ECG to the end of left ventricular outflow by PW, as aforementioned. The second interval was between the time from the top of the R-wave on ECG to the D point of the mitral valve by M-mode echocardiography at the parasternal long-axis view, and the time difference was calculated by subtracting the former time from the latter time.

In the CW, PW, and IIs-M-mode methods, the Dual Gate Doppler method and IIs-PW method, the IVRT was measured in the same heartbeat, whereas in the other methods, it was measured at two different heartbeats and subtracted. One professionally qualified technician recorded and performed the time-phase analysis at 200 mm/s, and each item was measured as the average of three heartbeats.

An additional study was performed to determine the cause of the difference in the IVRTs between the CW and PW methods. We compared the time from the R-wave on ECG to the aortic valve closure phase and from the R-wave on ECG to the mitral valve opening phase in 39 patients (mean age, 59 ± 22 years, 18 women) using the CW and PW methods, respectively.

### Statistical analysis

Values are expressed as mean ± standard deviation. The Spearman correlation coefficient, Wilcoxon signed rank-sum test, intraclass correlation, and Bland–Altman analysis were used as appropriate. Statistical analysis was performed using EZR version 1.54. In all analyses, *p*-values < 0.05 were considered statistically significant.

## Results

### Comparisons of IVRTs measured by different methods

The mean IVRT values for each method are listed in Table 1. According to the Wilcoxon signed rank sum test, only the PW time difference method (*p* = 0.984) showed no significant difference compared to the Dual Gate Doppler method.

**Table 1:** Average of isovolumic relaxation time and the results of Wilcoxon signed rank sum test

Method (*n* = 104)	Mean value ± SD (msec)	Significant probability
Dual Gate Doppler method	82.31 ± 22.19	–
CW method	92.51 ± 21.17	*p* < 0.0001
PW method	84.26 ± 22.06	*p* = 0.012
IIs-PW method	74.26 ± 20.00	*p* < 0.0001
IIs-M mode method	84.38 ± 22.00	*p* = 0.045
PW time difference method	82.60 ± 24.55	*p* = 0.984
M-mode time difference method	94.55 ± 25.70	*p* < 0.0001

*CW* continuous wave Doppler, *PW* pulsed Doppler, *IIs* the second sound in heart sound, *SD* standard deviation

**Fig. 2:**
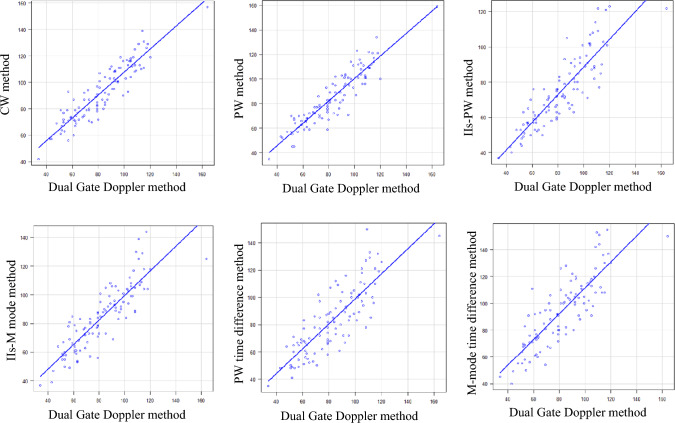
Correlations between the Dual Gate Doppler and each of the other methods

The correlations between the Dual Gate Doppler method and each of the other methods were all good, with Spearman correlation coefficients of 0.905 for the CW method, 0.914 for the PW method, 0.887 for the IIs-PW method, 0.881 for the IIs-M mode, 0.833 for the PW time difference method, and 0.837 for the M-mode time difference method (Fig. 2).

**Fig. 3:**
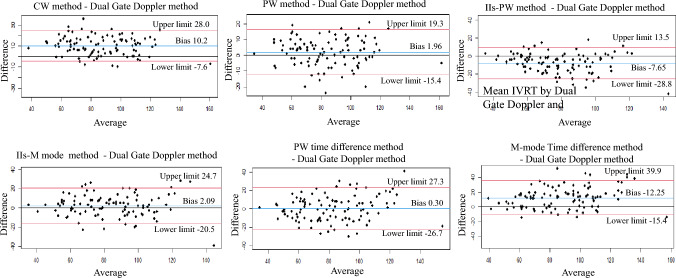
Bland–Altman plots comparing the isovolumic relaxation time measured using the Dual Gate Doppler method and the other methods

According to the Bland–Altman analysis, the methods showing small bias were the PW method, IIs-M mode and PW time difference methods. The latter two methods showed results free of errors in terms of additional and proportional errors (Table 2, Fig. 3).

**Table 2:** Results of Bland–Altman analysis for measuring the isovolumic relaxation time

	Addition error		Proportional error		
	95% confidence interval	Error or not	slope of the regression curve		Error or not
CW method	8.44–11.98	+	– 0.11	*p* = 0.25	–
PW method	0.24–3.68	+	0.02	*p* = 0.88	–
IIs-PW method	– 9.75– 5.56	+	– 0.21	*p* = 0.03	+
IIs-M mode method	– 0.15–4.37	–	– 0.02	*p* = 0.86	–
PW time difference method	– 2.39–2.98	–	– 0.18	*p* = 0.06	–
M-mode time difference method	9.50–15.00	+	– 0.26	*p* = 0.008	+

*CW* continuous wave Doppler, *PW* pulsed Doppler, *IIs* second sound in heart sound

### CW method versus PW method for measuring the IVRT

**Fig. 4:**
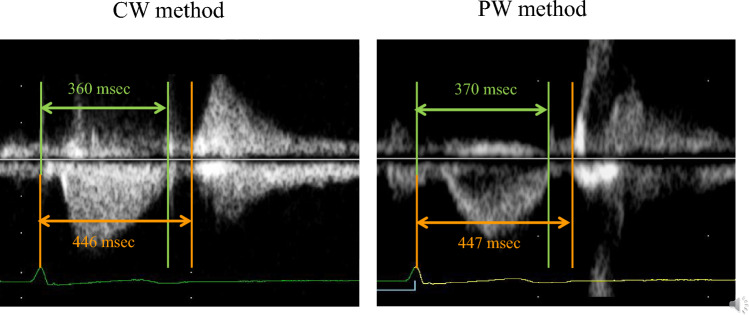
Left ventricular (LV) outflow and LV inflow by the continuous wave Doppler method (CW) and pulsed Doppler (PW) method in a representative case. The times from the top of the R-wave on electrocardiography to the end of LV outflow (arrow of green lines) and the beginning of LV inflow (arrow of orange lines) of each method were measured. The isovolumic relaxation time is calculated by the difference of each time

The IVRT measured by the CW method was longer than that measured by the Dual Gate Doppler method (10 ms) and PW method (12 ms). The mean interval times from the top of the R-wave to the beginning of mitral inflow were 446 ± 52 ms using the CW method and 447 ± 41 ms using the PW method, with no significant difference (Table 3). However, the interval time from the top of the R-wave on ECG to the end of left ventricular outflow was significantly shorter in the CW method than in the PW method (360 ± 28 ms versus 370 ± 30 ms, *p* = 0.007). Left ventricular outflow and inflow in one case are shown in Fig. 4. In this case, the IVRT in the CW method was 86 ms, which was longer than that in the PW method.

**Table 3:** Results of the IVRT in CW method and PW method

	PW method (msec)	CW method (msec)	*p*
Interval time from the top of the R-wave to the end of left ventricular outflow	370	360	0.007
Interval time from the top of the R-wave to the beginning of mitral inflow	447	446	0.397

*PW* pulsed Doppler, *CW* continuous wave Doppler

## Discussion

In the present study, all other methods showed good correlations compared to the Dual Gate Doppler method for the estimation of IVRT. However, the IVRT showed slightly different values depending on the method used. Of these, the PW method was the simplest and most practical method to measure the IVRT.

### The most reliable method for measuring the IVRT

In our study, only the PW time difference method showed no significant difference compared to the Dual Gate Doppler method by the Wilcoxon signed rank test. In the PW time difference method and the PW method, we can determine the aortic valve closure phase and mitral valve opening phase simply using only PW, which is the same as the Dual Gate Doppler method. In the PW time difference method, each sample volume can be set at the correct site as indicated by the guidelines. On the other hand, in the PW method, we need to set the sample volume at the site, where both of LV outflow and inflow waveforms can be depicted simultaneously. Therefore, the PW time difference method can depict pulsed Doppler waveforms more clearly compared to the PW method. However, the PW time difference method cannot be used in patients with arrhythmias because the RR interval is significantly different for each individual heartbeat. Other methods using PCG and M-mode echocardiography indirectly determine the phase and measurements become complicated. Overall, the PW method is the easiest to utilize for measuring the IVRT.

### Accuracy of the methods using PW compared to that using CW

The IVRT measured using the CW method was significantly longer than that measured using the Dual Gate Doppler method. This was attributed to the fact that the duration of left ventricular outflow measured by the CW method was significantly shorter than that measured by the PW method by approximately 10 ms. Thus, the IVRT was prolonged. The reason is a technical problem in that the PW method allows fine adjustment of the sample volume to the left, right, up, and down, and the identification of the end and start points in the two directions of left ventricular outflow and inflow is easy; conversely, the CW method limits fine beam adjustment to only the left and right. Therefore, PW should be used to determine the aortic valve closing and mitral valve opening phases rather than CW.

### Effect of determining the timing of aortic valve closure by IIs

The IIs-PW method had a significantly shorter IVRT than the Dual Gate Doppler method. IIs is caused by the rebound of blood in the great vessels, which is abruptly stopped by the closing of the semilunar valve. Immediately after aortic valve closing, structures of the aortic root, such as the semilunar valve and arterial wall, oscillate and generate IIs [10]. As a result of the time lag in the aforementioned process, the beginning of IIs is slightly delayed to aortic valve closure, and the IVRT becomes shorter.

In addition to the mechanism, the heart sound waveform recorded by the ultrasound system tends to generate noise due to the position of the microphone, the examinee’s physique, and other unknown factors, which may cause a delay in IIs.

### Effect of determining the timing of mitral valve opening by M-mode echocardiography

In the M-mode time difference method, the analysis showed the worst results compared to the other methods. In this method, the D point of the mitral valve trace was recorded by M-mode echocardiography, which indicated that the timing of mitral valve opening was delayed compared to the beginning of mitral inflow in the Dual Gate Doppler method. The delay of the D-point on the mitral valve trace is owing to the through-plane phenomenon [11] of the M-mode method. In other words, the M-mode beam may not accurately capture the mitral valve motion of the leaflet tip. As a result, point D may shift later than the true mitral valve opening phase.

## Limitations

This study has several limitations. First, this was a single-center prospective study. Because of the small number of participants, it was not possible to classify participants by disease or status and estimate the IVRT. We need to substantiate this study’s results in a large population or multicenter studies. Second, time-phase analysis using heart sounds requires a precise and noise-free heart sound microphone. We used a sensitive microphone, however, the waveform was affected by the participants’ physique. Now that echocardiography has been performed without a microphone, it may be difficult to expect a high-quality microphone for such evaluation. However, the time-phase analysis of cardiac function is highly reproducible, useful, and convenient. Dual Gate Doppler is easy to measure and is useful for evaluating cardiac hemodynamics [12]. We would like to continue to study the usefulness of echocardiography using time-phase analysis.

## Conclusions

In the present study, the PW method was the simplest and most practical method to measure the IVRT when the Dual Gate Doppler method cannot be used. We need to recognize the differences and characteristics of the measurement methods for evaluation of the IVRT and accurately estimate diastolic function.
